# Questioning the Ubiquity of Neofunctionalization

**DOI:** 10.1371/journal.pcbi.1000252

**Published:** 2009-01-02

**Authors:** Todd A. Gibson, Debra S. Goldberg

**Affiliations:** 1 Computational Bioscience Program, University of Colorado Denver, Aurora, Colorado, United States of America; 2 Department of Computer Science, University of Colorado Boulder, Boulder, Colorado, United States of America; Department of Ecology and Evolution University of Chicago, United States of America

## Abstract

Gene duplication provides much of the raw material from which functional diversity evolves. Two evolutionary mechanisms have been proposed that generate functional diversity: neofunctionalization, the de novo acquisition of function by one duplicate, and subfunctionalization, the partitioning of ancestral functions between gene duplicates. With protein interactions as a surrogate for protein functions, evidence of prodigious neofunctionalization and subfunctionalization has been identified in analyses of empirical protein interactions and evolutionary models of protein interactions. However, we have identified three phenomena that have contributed to neofunctionalization being erroneously identified as a significant factor in protein interaction network evolution. First, self-interacting proteins are underreported in interaction data due to biological artifacts and design limitations in the two most common high-throughput protein interaction assays. Second, evolutionary inferences have been drawn from paralog analysis without consideration for concurrent and subsequent duplication events. Third, the theoretical model of prodigious neofunctionalization is unable to reproduce empirical network clustering and relies on untenable parameter requirements. In light of these findings, we believe that protein interaction evolution is more persuasively characterized by subfunctionalization and self-interactions.

## Author Summary

Molecular evolution studies have shown that the redundancy intrinsic to gene duplication may allow one gene duplicate to acquire a new function (neofunctionalization) or for both duplicates to each assume a subset of the ancestral gene's functions (subfunctionalization). Studies of networks of interacting proteins and models of evolving protein interaction networks have shown that both subfunctionalization and neofunctionalization are widespread in protein evolution. Here, we present evidence that shows that the methods and models that have established neofunctionalization as a ubiquitous force in protein interaction network evolution are flawed and under reexamination support subfunctionalization, not neofunctionalization. We start by reviewing the methods and models that engender prolific subfunctionalization and neofunctionalization in evolution. We then critically approach neofunctionalization. We show that biases in protein interaction assays, failure to consider concurrent and subsequent gene duplications in evolutionary inferences, and an inability of theoretical models to reproduce empirical clustering have all led to neofunctionalization being erroneously identified as a pervasive force in evolution.

## Introduction

Gene duplication is readily accepted as a primary mechanism for generating organismal complexity. Phenomena proposed for the fate of gene duplicates include neofunctionalization and subfunctionalization. Neofunctionalization posits that the functional redundancy intrinsic to initially identical gene duplicates releases one duplicate from selective pressure. While under neutral selection one of the duplicates can accumulate random mutations and potentially acquire novel and beneficial functions [1]. Subfunctionalization states that both gene duplicates acquire mutations resulting in each duplicate assuming a complementary subset of the ancestral gene's original functions [2].

Gene duplication and subsequent neofunctionalization and subfunctionalization have straightforward analogs in models of protein interaction network (PIN) evolution. With proteins as nodes, edges between proteins represent physical interactions and serve as an indication of protein function. Proteins with identical sets of interacting partners are presumed to have identical functions. Gene duplication is modeled by copying a protein node in the network along with its interactions. Neofunctionalization and subfunctionalization are modeled by the gain and loss of interactions respectively. This straightforward representation has made PINs an attractive target for the study of evolution.

Both neofunctionalization and subfunctionalization have been shown to occur in protein interaction analyses of extant species. Since paralogs are by definition related by gene duplication, the similarities and differences between the interactions of paralogous pairs have been used to elucidate the role of neofunctionalization and subfunctionalization in the fate of gene duplicates.

**Figure 1:**
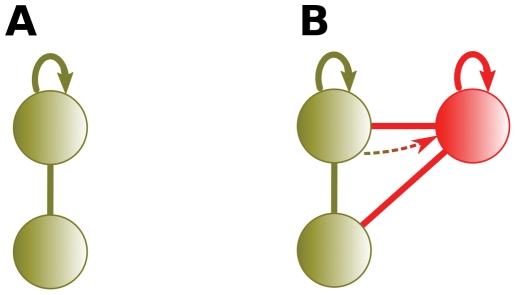
Duplication of self-interacting proteins. (A) An interaction between a protein and a self-interacting protein. (B) When the self-interacting protein duplicates, the duplicates interact.

Wagner [3],[4] noted that an interaction between a paralogous pair forms by one of two methods: either the duplication of a self-interacting protein (Figure 1), or a de novo interaction forming between the pair sometime after duplication.

Wagner's analysis of three Saccharomyces cerevisiae interaction datasets revealed that the vast number of interacting duplicate pairs were not themselves self-interacting. Therefore, the absence of homomeric interactions in interacting paralogous pairs suggested that these interactions formed de novo (i.e., neofunctionalization). Extrapolating the probability of an interacting paralogous pair to the entire network, Wagner estimated that Saccharomyces cerevisiae adds between 108 and 294.5 interactions de novo every million years.

Wagner also compared the age of paralogs to the number of shared interaction partners. Wagner found that, except for the most-recently duplicated genes, duplicate pairs have lost on average from 85 to more than 90 percent of their shared interactions depending on their age and the dataset examined [4]. The rapid loss of common interacting partners between duplicates strongly suggests that subfunctionalization occurs quickly after duplication. A more recent study using similar methods measured 93% shared interaction loss in yeast [5].

**Figure 2:**
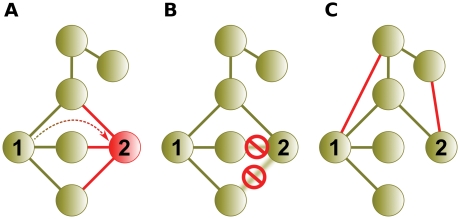
He and Zhang [6] illustrate the presence of neofunctionalization through interaction data analysis. (A) Paralogous proteins 1 and 2 initially share all 3 interacting partners. (B) In the absence of neofunctionalization, the number of interacting partners should remain at 3 as redundant interactions are lost over time. He and Zhang show that the number of interacting partners increases as the age of paralogs increases. (C) The increase in interacting partners is attributed to neofunctionalization (i.e., the de novo gain of interactions).

He and Zhang also found evidence of rapid subfunctionalization followed by a prolonged period of neofunctionalization in Saccharomyces cerevisiae protein interactions [6]. They reasoned that the set of nonredundant interacting partners shared between paralogous pairs should remain constant over time if subfunctionalization occurs without neofunctionalization. They ascertained that the set of nonredundant partners increased with the age of the paralogous pair, indicating the presence of neofunctionalization (Figure 2).

Neofunctionalization and subfunctionalization also appear in theoretical models of protein interaction evolution. The first model combining both neofunctionalization and subfunctionalization came from Solé and colleagues in 2002 [7]. Their duplication and diversification model iteratively duplicates a random gene and its interactions, followed by probabilistically deleting copied interactions (subfunctionalization) and adding new interactions (neofunctionalization). A number of topological measures were found to be consistent between both the network produced by their model and observed Saccharomyces cerevisiae protein interactions, including connectivity, clustering coefficient, power-law degree exponent, and path length.

## Results

Despite prevailing theory which identifies neofunctionalization as a prominent force in the evolution of protein interactions, here we demonstrate that subfunctionalization and self-interactions sufficiently and more simply explain results previously attributed to neofunctionalization. While others have promoted the viability of subfunctionalization and the role of self-interactions in gene duplication, they have not challenged the putative ubiquity of neofunctionalization with a contrarian argument [8],[9]. We now describe in detail the effect underreported data in proteomic assays, misinterpreted interaction data, and model topology have had on the analyses and models which promote ubiquitous neofunctionalization.

### Underreported Yeast Self-Interactions

**Figure 3:**
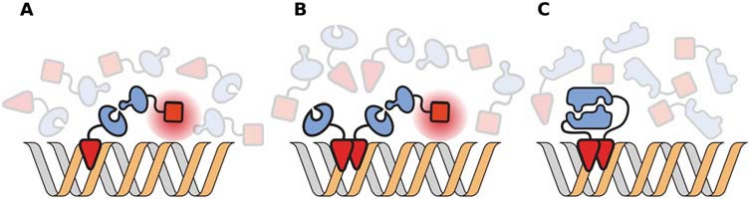
Shortcomings of the yeast two-hybrid assay. (A) The traditional view of the yeast two-hybrid assay. A bait protein is hybridized with the GAL4 binding domain which binds to the upstream activation sequence for galactose (UAS_G_). A prey protein hybridized with the GAL4 activation domain interacts with the bait protein. The complex forms a functional transcriptional activator and the downstream reporter gene is expressed. (B) A more accurate view of yeast two-hybrid assay. The GAL4 binding domain actually binds to UAS_G_ as a dimer. (C) If the GAL4 binding domain is hybridized to a self-interacting protein, self-interacting protein bait dimerizations would reduce the probability of bait-prey interactions.

The two most common high-throughput assays used to determine yeast protein interactions, yeast two-hybrid (Y2H) assays and affinity purification with mass spectrometry (AP-MS), have limited ability to discern self-interactions. In Y2H assays, self-interacting baits interact together and self-interacting prey interact together reducing the concentration of bait/prey interactions with respect to their heterointeracting counterparts. Additionally, the GAL4 binding domain binds DNA as a dimer [10],[11], allowing homomeric bait pairs to dimerize with each other instead of prey (Figure 3) [12],[13].

Large-scale TAP-MS studies [14]–[16] report no homomeric interactions due to a lack of endogenous (untagged) homomeric mates to discern from the affinity tagged protein [17]. Other large-scale AP-MS studies [18] use small epitope tags. The epitope tagged homomer very nearly overlaps with its endogenous mate in the MS spectra making the flagged homomer difficult to discern from its unflagged mate. For example, only a single homomeric interaction among 3,617 reported interactions was identified by Ho and colleagues in 2002 [18] using the FLAG epitope tag.

Examination of the physical data supports a higher proportion of homomers than yeast two-hybrid and AP-MS studies indicate. First we compiled a set of non-redundant structures containing Saccharomyces cerevisiae protein complexes from The Protein Data Bank (PDB) [19]. We then cross-referenced these structures to the iPfam database of PDB protein interactions [20] (see Methods). A tally of identical proteins self-interacting across different polypeptide chains confirms the ubiquity of self-interacting proteins. There are 207 non-redundant yeast structures containing 210 Saccharomyces cerevisiae proteins, 149 of which (71%) are self-interacting. Similarly, the BRENDA enzyme database [21] contains 102 Saccharomyces cerevisiae enzymes with specific hetero- and homomeric k-mer counts (monomer, dimer, trimer, etc.). Self-interacting enzymes (k-mers with *k*≥2) accounted for 60% of the Saccharomyces cerevisiae enzymes. At the protein complex level, Pereira-Leal et al. [22] found that 90% of the structures in the Protein Quaternary Structure database [23] include homomeric interactions, and other studies also identify a high proportion of homomeric interactions [8],[9]. By contrast, in high-throughput yeast two-hybrid studies by Uetz et al. and Ito et al. detected homomeric proteins in only 4.6% and 6.6% respectively of the proteins included in their core interaction sets.

Additional evidence supports widespread duplication of self-interacting proteins. Zhang et al. found that, of nine tested attributes, homology was one of four attributes showing substantial predictive value for predicting co-complexed pairs of proteins [24]. Additionally, interactions within paralogous families are much more likely than within randomly-formed families (*P*<10^−6^, see Methods). The wide disparity between the frequency of paralogous versus random interactions indicate that some process other than the random, de novo addition of interactions which characterize neofunctionalization is at work. Duplication of homomers is a more parsimonious explanation than neofunctionalization for the interaction evolution between paralogous proteins.

Underrepresented self-interactions in interaction data have not been previously realized, leading to erroneous assertions. Wagner [4] identified 31 interacting paralogous pairs from Y2H assays (gathered from Uetz et al., and Ito et al. [25],[26]), and 13 interacting paralogous pairs from non-Y2H assays (gathered from MIPS [27]). In 34 of these 44 interacting paralogous pairs, neither protein of the pair had a self-interaction. Looking for an evolutionary explanation for the presence of the 34 paralogous interactions, Wagner reasoned that either the 34 paralogous pairs (i.e., 68 proteins) lost their ability to self-interact, or that the 34 interactions appeared de novo sometime after duplication. Wagner concluded that the most parsimonious explanation was 34 interactions gained de novo, rather than 68 lost self-interactions. This reasoning led Wagner to postulate that of the other combinations of self- and paralogous- interacting pairs, de novo interaction gain accounted for all but two pairs in which both protein members self-interacted and interacted with each other (as in Figure 1B).

Using the number of putative de novo gains as a metric, Wagner extrapolated to arrive at the ubiquitous 108–294.5 de novo interactions gained per million years of evolution. Once assay biases are considered as an alternative to evolutionary loss in explaining the absence of self-interactions among Wagner's paralogous pairs, the opposite conclusion is reached: paralogous interactions are more parsimoniously explained by duplicating homomers, not de novo interaction gain.

### Concurrent Gene Duplication and Subfunctionalization

Complementary degenerative mutations intrinsic to subfunctionalization take the form of complementary interaction loss in its network analog. One interaction from each pair of redundant interactions may be lost, but He and Zhang [6] reasoned that in the absence of neofunctionalization, the union of the duplicates' interacting partner sets will remain unchanged over time. Figure 2A features a portion of the methodology used by He and Zhang to test this. They compared the ages of gene duplicate pairs to the union of their interacting partner sets. Contrary to what they believed subfunctionalization alone would show, they found that the union size increased with the age of the duplicate pair. Neofunctionalization was credited with the increase in the number of interacting partners.

**Figure 4:**
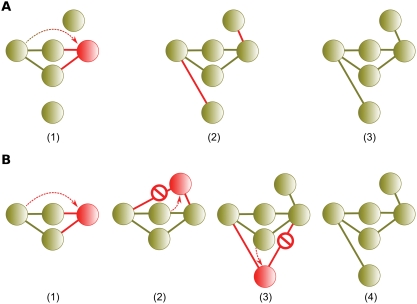
Neofunctionalization vs. concurrent gene duplication and subfunctionalization. (A1) Gene duplication. Shown also are two additional proteins elsewhere in the network. (A2) According to He and Zhang (2005), additional interactions gained by paralogous pairs over time are explained by the formation of de novo interactions. (A3) The resulting network. (B1) Gene duplication. (B2) An interacting partner duplicates, including the loss of a redundant interaction. (B3) Another partner duplicates and loses a redundant interaction. (B4) The resulting network is indistinguishable from that postulated for neofunctionalization.

This argument fails to recognize that the interacting partners evolve as well. Gene duplication and subfunctionalization occur among all genes concurrently with the paralogous protein pair under study. Figure 4A shows a typical gene duplication scenario followed by neofunctionalization as proposed by He and Zhang. Figure 4B shows that the increase in interaction partners over time attributed to neofunctionalization is readily explained by gene duplication occurring elsewhere in the network. After gene duplication, each additional interacting partner acquired by the duplicate pair over time may simply result from an interacting partner undergoing gene duplication.

**Figure 5:**
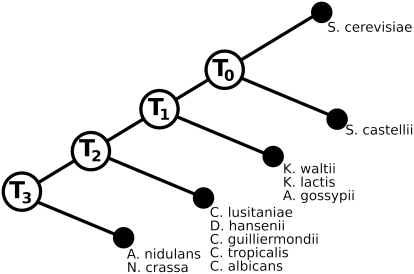
A fungal phylogenetic tree showing ancestral species nodes into which Saccharomyces cerevisiae duplicates are grouped (*T*_0_–*T*_3_). Groupings were generated from gene trees reported in reference [28]. Ancient duplications occurred in ancestral node *T*_3_ and the most recent duplications occurred in *T*_0_.

We validated the role subsequent duplications play in increasing the number of interacting partners by counting interacting partners of gene duplicates both before and after accounting for subsequent duplications. Saccharomyces cerevisiae gene duplicates were binned into four different age groups based on genome-wide gene trees developed from 19 fungal genomes (drawn from revised data provided on Web site associated with Ref. [28], see Methods). Figure 5 shows the phylogenetic nodes which correspond to the age bins gene duplicates were placed into.

**Figure 6:**
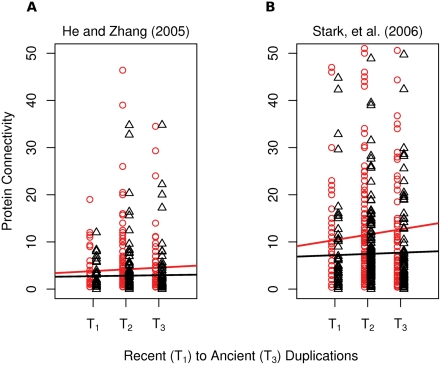
Change in the number of interacting partners (protein connectivity) over time. Proteins are aligned with the phylogenetic period from Figure 5 in which they were born (see Methods). Red circles identify the connectivity of gene duplicates born at the indicated phylogenetic timepoint: *T*_1_, *T*_2_, and *T*_3_. The red trend line indicates that the connectivities of gene duplicates increase over time. Black triangles identify the same proteins after removing interactions with more recent duplicates. The black trend line indicates that once subsequent duplications are accounted for, the connectivities of paralogous genes remain largely unchanged. This is consistent with the alternate explanation proposed in Figure 4B. (A) The combined interaction datasets [29],[30] used by He and Zhang [6]. (B) Physical interactions from BioGrid [31].

Interacting partners of gene duplicates were then tallied and plotted according to their age bin (Figure 6). Before considering subsequent duplications, the number of interacting partners of gene duplicates increases with the age of the duplicate, consistent with the findings of He and Zhang [6]. Once interactions associated with subsequent gene duplications are removed, interacting partner counts show little change over time (see Methods).

Another observation is that under concurrent gene duplication, the interacting partners of a duplicate pair should be enriched in paralogs born of subsequent duplications. This is illustrated in Figure 4B. The four interacting partners in frame B4 are two pairs of paralogs which arose via gene duplications subsequent to the original duplication in frame B1. We sought this evidence in the interacting partners of each duplicate pair present in the both combined datasets [29],[30] used by He and Zhang [6] and the physical interactions from BioGrid [31] (see Methods). As we expected, the interacting partners of duplicate pairs are significantly enriched with paralogs born of subsequent duplications. The mean proportion of interacting partners which are paralogous in the He and Zhang dataset is 0.029 (*P*<10^−6^, random expectation 0.0014) and 0.042 (*P*<10^−6^, random expectation 0.0025) in the BioGrid data.

### Evolutionary Models

Theoretical models of PIN evolution reproduce characteristics of observed interaction networks while honoring aspects of biological evolution. In 2002, Solé et al. introduced a “duplication and diversification” model which established the relevance of gene duplication and interaction gain and loss to PIN evolution [7]. The following year Vázquez and colleagues published an alternative model of PIN evolution which includes interaction loss due to subfunctionalization, but does not include neofunctionalization [32]. The common feature of both models is subfunctionalization. That is, both models include a parameter specifying the probability of losing (or retaining) interactions to protein partners shared by both the progenitor and progeny genes. The models differ in the method through which new interactions are formed in the network. A second parameter of the Solé et al. model controls the probability of forming new interactions from the newly duplicated gene to each extant gene in the network. A second parameter of the Vázquez et al. model controls the probability of forming a new interaction from the newly duplicated gene to the progenitor gene. Essentially, the difference between these two models can be characerized as neofunctionalization versus homomeric duplication (i.e., duplicating a self-interacting gene). This difference reflects the dichotomy we've established and therefore deserve additional attention.

We have quantified this dichotomy using the topological measure *C*, the clustering coefficient [33]:
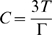

*T* is the number triangles (three fully-connected nodes), and Γ is the number of connected triples (a node connected to an unordered pair of other nodes).

The clustering coefficient is a relavant measure for two reasons. First, gene duplications, subfunctionalization, neofunctionalization, and homomeric duplication each produce a measurable change in the number of triangles and connected triples which comprise the clustering coefficient. Second, protein interaction networks have been found to have high clustering coefficients relative to random networks [3], [7], [34]–[36]. Table 1 shows that the clustering coefficients for several Saccharomyces cerevisiae datasets are a factor of 5, 10, and more above that of equivalent random networks. We seek to identify those evolutionary events which contribute to a high clustering coefficient.

**Table 1:** Network measures, including *C*, the clustering coefficient of Saccharomyces cerevisiae protein interaction networks.

Nodes	Edges	Triangles	Connected Triples	*C*	*C*_random_	*C*/*C*_random_	Citation
4674	14294	16821	431696	0.117	0.029	4.0	[6]
1040	1040	3017	34006	0.266	0.040	6.7	[37]
5055	41338	122215	2074478	0.177	0.029	6.1	[31]
4008	9857	8851	180732	0.147	0.015	9.8	[38]
2406	5244	5441	39288	0.415	0.005	83.0	[39]
1642	9100	63084	306505	0.617	0.060	10.3	[40]

Saccharomyces cerevisiae exhibits clustering dramatically greater than equivalent random networks. Protein interaction networks were constructed from various experimental, curated, and high-confidence Saccharomyces cerevisiae protein interaction datasets as cited. The mean clustering coefficient of equivalent random networks, *C*_random_, was calculated as described in Methods.

**Figure 7:**
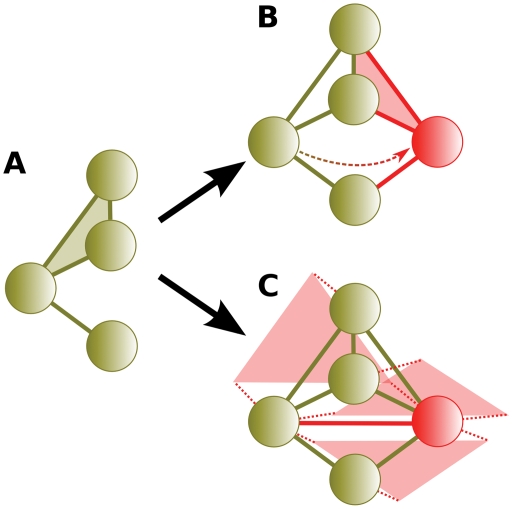
Triangles and connected triples in gene duplication. (A) The network has *T* = 1 triangle and Γ = 5 connected triples. (B) Simple duplication adds a duplicate of the progenitor's single triangle to the network. There are *γ_p_* = *k_p_*(*k_p_*−1)/2 = 3 connected triples centered around the progeny, and an additional Σ*k*_g_ = 5 connected triples centered on the neighbors. (C) If the progenitor is self-interacting, an additional edge between the progenitor and progeny is formed, thus increasing the simple duplication counts by *k_p_* = 3 additional triangles (extruded for clarity) and 2*k_p_* additional connected triples (the progenitor and progeny are both centered on *k_p_* additional connected triples due to the dimerizing interaction).

**Figure 8:**
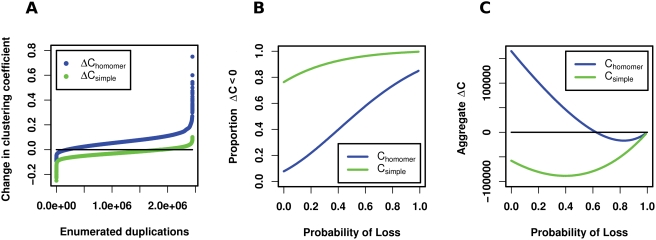
The effect of gene duplication on the clustering coefficient. Every connected network containing three to nine nodes was enumerated producing 273,191 networks containing 2,445,434 nodes. (A) Changes to the clustering coefficient resulting from simple duplication and homomeric duplication. Each of the 2,445,434 nodes was duplicated twice, once as self-interacting (homomeric) and once as non-self-interacting (simple). Shown is the change in clustering coefficient for each duplication, ordered by magnitude. The enumerated networks serve as possible subnetworks of larger protein interaction networks. The magnitude of the vertical axis is determined by the size of the network, but the shape of the curves around zero remains unchanged. (B) The severe effect subfunctionalization has on the clustering coefficient. The vertical axis represents the portion of the 2,445,434 gene duplications in the enumerated networks which result in a decrease in the clustering coefficient. Probability of Loss is the probability the gene duplicate (progeny) loses each of its interactions due to subfunctionalization. Even without losses suffered due to subfunctionalization, simple duplications reduce the clustering coefficient in over 76% of examined duplications. By contrast, clustering coefficients produced via homomeric duplication are far more likely to increase even in the face of interaction losses caused by subfunctionalization. (C) The effect of subfunctionalization on aggregate Δ*C*. The change in clustering coefficient aggregated for all 2,445,434 duplications at each loss probability. While aggregate Δ*C* of simple duplication is below zero for all loss probabilities, homomeric duplications remain above zero until the Probability of Loss≈0.62.

The change in clustering coefficient resulting from simple gene duplication, Δ*C*_simple_ (i.e., duplicating a node and its interactions without regard to subsequent interaction loss), occurs locally. The change can be defined in terms of the progenitor's (*p*) triangles (*t_p_*) and degree (*k_p_*), and the degree of the progenitor's neighbors (*k*_g_,g = 1..*k_p_*, see Figure 7). Because Δ*C*_simple_ is restricted to the neighborhood around the duplication progenitor, the majority of duplication scenarios can be modeled by considering only small subnetworks. We enumerated all connected networks (i.e, all non-isomorphic networks with a single component) having three to nine nodes. This produces 273,191 networks containing a total of 2,445,434 nodes. Each node in every network was duplicated and the clustering coefficient before and after was measured. In 1,864,851 (over 76%) of the possible duplications Δ*C*_simple_<0 (Figure 8A). In other words, most simple gene duplications decrease the clustering coefficient. Table 2 shows the change in clustering coefficient for enumerated networks as the number of nodes considered increases.

**Table 2:** Δ*C* as the number of nodes in the enumerated networks increases.

		Δ*C*_simple_/Δ*C*_homomer_		
Nodes	Possible Duplications	Fraction<0	Fraction>0	Fraction = 0
= 3	6	0.500/0.000	0.000/0.500	0.500/0.500
≤4	30	0.500/0.000	0.000/0.733	0.500/0.267
≤5	135	0.593/0.037	0.037/0.859	0.370/0.104
≤6	807	0.685/0.055	0.082/0.911	0.233/0.035
≤7	6778	0.749/0.065	0.146/0.922	0.106/0.013
≤8	95714	0.765/0.072	0.190/0.922	0.044/0.006
≤9	2445434	0.763/0.078	0.221/0.919	0.017/0.004

As the number of nodes increases in the enumerated networks the probability that a duplication reduces (increases) Δ*C* converges. Shown are probabilities for both simple duplication and homomeric duplication (duplication of a self-iteracting node).

We then incorporated a complementary loss probability into our simple gene duplications in the enumerated networks to quantify the impact subfunctionalization has on the clustering coefficient. Subfunctionalization generates an even greater proportion of duplications reducing the clustering coefficient. Figure 8B and 8C show the effect subfunctionalization has on the clustering coefficient in the enumerated networks.

**Figure 9:**
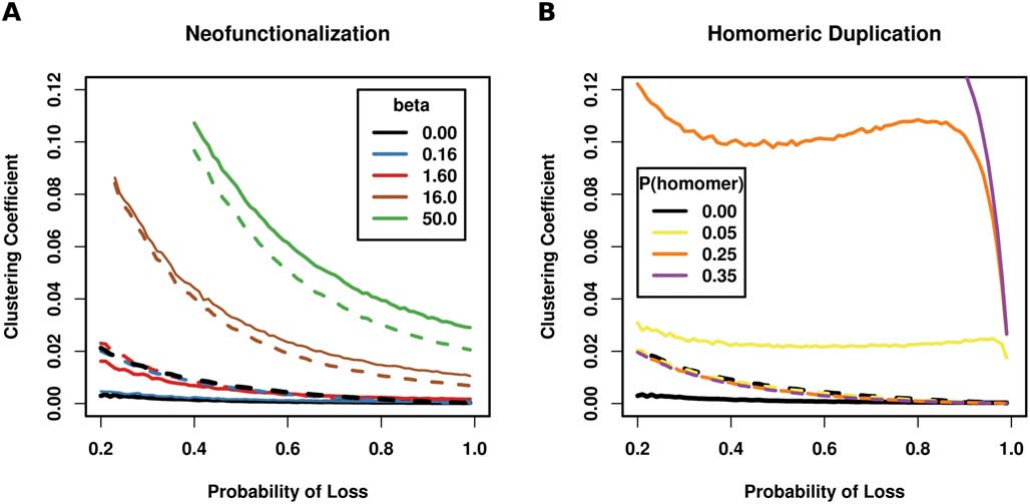
The clustering coefficient of networks featuring simple duplication, neofunctionalization, subfunctionalization, and homomeric duplication. Each plot shows the clustering coefficient for different probabilities of a gene duplicate losing a redundant interaction (i.e., different levels of subfunctionalization). Lines are grouped into pairs by color. A solid line is a model with a specific parameter, and a dashed line of the same color is the model's random equivalent (see Methods). The black line pairs represent simple duplication and subfunctionalization (i.e., no neofunctionalization or homomeric duplication) and are therefore identical in both plots. (A) The Solé et al. model which includes neofunctionalization [7]. (B) Homomeric duplication as found in the Vázquez et al. model [32].

The preponderance of enumerated network duplications which reduce the clustering coefficient suggest that additional evolutionary mechanisms beyond that produced by simple gene duplication and subfunctionalization are required to achieve a high clustering coefficient. Indeed, the black lines in Figure 9 show that networks evolved via simple duplication and different degrees of subfunctionalization produce clustering coefficients lower than their random equivalents. The high clustering coefficients relative to equivalent random networks observed in empirical data are unattainable using a simple duplication and subfunctionalization network model.

Solé et al. extend simple duplication and subfunctionalization by adding a probability *α* of adding a de novo interaction from a gene duplicate to each of the existing genes in the network. This probability is defined as: 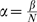 where *N* is the number of nodes currently in the network and *β* is a constant reflecting the expected number of de novo interactions added to each gene duplicate [7] (see Discussion). The value of *β* (that is, the frequency of neofunctionalization) can be selected to achieve any desired clustering coefficient. In the extreme, new interactions could be added exhaustively driving the clustering coefficient towards one, that is, the clustering coefficient of a completely-connected network. However, the neofunctionalization model adds random interactions, which drives the clustering coefficient towards random expectation. We updated our simple duplication and subfunctionalization model to include neofunctionalization as implemented in the Solé et al. model. Figure 9A shows the model for various values of *β*. Biologically plausible *β* generate too few new interactions and are unable to appreciably affect the topology of the simple duplication model. The value of *β* derived in Solé et al. [7] is 0.16. The resulting clustering coefficient (blue line) is indistinguishable from the simple duplication model. At *β* = 1.6 (red line), the networks and their random equivalents are nearly the same. Increasing *β* to 16 and 50 (brown and green lines respectively) increases the clustering coefficient but also increases the clustering coefficient of its random equivalent. These extreme values for *β* highlight the close relationship between the neofunctionalization model and its random equivalent. The random edges inherent to neofunctionalization drive the clustering coefficent toward random expectation. At *β* = 16 and *β* = 50, each gene duplicate adds an average of 16 and 50 additional interactions respectively which is biologically untenable.

In order to achieve higher clustering coefficients, additional triangles must be added to the network while minimizing the number of triples added to the network. Gene duplication alone can increase the number of triangles if the duplicate is a self-interacting protein. Figure 8A shows that a self-interacting protein increases the clustering coefficient (Δ*C*_homomer_>0) of the enumerated networks in 2,246,876 (almost 92%) of possible duplications. In fact, Δ*C*_homomer_ is always greater than Δ*C*_simple_ for an equivalent duplication (a proof of this can be found in the Supporting Information, Text S1).

To contextualize the ability of homomeric duplication to increase the clustering coefficient, we updated our simple duplication model to include homomeric duplication as defined in Vázquez et al. [32]. Note that Vázquez et al. use the term *heteromerization*. Figure 9B shows that the model produces clustering coefficients markedly higher than those of their random equivalents. It is notable that the probabilities sampled for Figure 9B produce substantially high clustering coefficients despite being much lower than the proportion of homomeric proteins we reported on earlier in structure and enzyme datasets (71% and 60% respectively) The structure and enzyme probabilities are omitted from Figure 8B simply because the higher clusterings they produce result in uninformative lines which are nearly vertical on the plot. Despite the increase in clustering coefficient due to homomeric duplication, the random equivalent networks remain virtually identical to the simple duplication random equivalent, reflecting the modest effect a single edge added by homomeric duplication has on the number of edges and hence on the expected (i.e., random) number of triangles.

Gene duplication, neofunctionalization, subfunctionalization, and homomeric duplication each uniquely affect the clustering coefficient. Only homomeric duplication achieves clustering coefficients appreciably higher than clusterings in equivalent random networks.

## Discussion

A variety of methods have been used to establish the ubiquity of neofunctionalization in protein interaction networks. For each of these we have identified very different factors which question of the ubiquity of neofunctionalizaiton. We now elaborate on our findings and identify broader implications of our results.

### Assay Biases

Biological network research is particularly sensitive to dataset biases [41]. Identified correlations between topology and essentiality have been challenged for relying on small-scale assay data which are more frequently the focus of interesting (i.e., essential) genes [42], and topological inferences of underlying networks have been questioned due to the incomplete sampling of biological assays provide [36],[43]. The dearth of homomeric interactions in data produced from Y2H and AP-MS assays is another bias which was not previously recognized and needs to be accounted for. The line of reasoning establishing the ubiquity of neofunctionalization was based on such biased data.

Wagner based his conclusions on an assumption that the lack of homomeric interactions was a true characteristic of the data. Failure to account for homomeric biases continues to affect evolutionary inferences. Recently, Presser et al. [44] determined that many more self-interacting proteins existed prior to the whole-genome duplication event (WGD) in Saccharomyces cerevisiae evolutionary history than are observed today. This determination was accompanied by a discussion about evolutionary causes underlying the loss of self-interactions from the WGD to today. Once the lack of self-interactions is recognized as a result of assay artifacts and not a true characteristic of the data, a simpler conclusion can be drawn: Saccharomyces cerevisiae had many self-interacting proteins prior to the WGD, and continues to have many self-interacting proteins today.

### Concurrent and Subsequent Duplication

**Figure 10:**
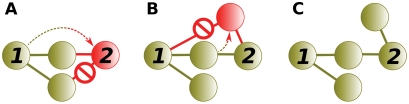
Underestimating the interaction conservation rate (equivalently, overestimating the interaction loss rate). The conservation rate is the number of shared interacting partners divided by the total number of partners. (A) Gene 1 is duplicated to create paralogous pair 1 & 2. The true conservation rate is 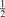. (B) A neighbor of the paralogous pair duplicates and loses a redundant interaction. (C) The network as observed. The paralogous conservation rate of 1 & 2 is erroneously underestimated to be 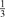. Equivalently, the true loss rate of 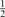 is overestimated to be 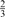.

Another line of reasoning establishing the ubiquity of neofunctionalization was based on the neighbor sets of duplicated proteins. When inference relies on the neighbors of protein duplicates, accurate estimates require recognizing that those neighbors are also subject to duplication. This omission resulted in He and Zhang's erroneous conclusions. He and Zhang are not alone in failing to recognize this. Concurrent and subsequent duplication has been universally ignored in estimating the rate of subfunctionalization, that is, the proportion of conserved interactions among gene duplicates [4], [5], [45]–[47] (Figure 10). The probability of interaction conservation is estimated by dividing the number of interacting neighbors of both members of a paralogous pair by the total number of neighbors between the pair. If the duplication event which produced the paralogous pair predates the duplication of any of its interacting neighbors, estimates of conservation of interactions are underestimated. Equivalently, estimates of interaction loss are overestimated (Figure 10).

### Theoretical Models

We found that simple duplication and subfunctionalization are unable to produce clustering coefficients observed in empirical protein interaction networks. Neofunctionalization is also ineffective at increasing the clustering coefficient unless untenably high values of *β* are used. The clustering coefficients resulting from these high values of *β* are bound closely to the clustering coefficients of random equivalent networks, contrary to observed networks. We found that producing high clustering coefficients with low clusterings in random equivalents as observed in empirical protein interaction networks requires the duplication of self-interacting proteins.

A discrepancy remains between our observations and those of Solé et al. [7]. They reported that at *β* = 0.16 their duplication and diversification model generated clustering coefficients consistent with Saccharomyces cerevisiae protein interaction networks. As illustrated in Figure 9A, we found that the same parameter value produces clustering coefficients much lower than observed protein networks and lower than equivalent random networks.

This discrepancy is resolved upon further examination. First, the clustering coefficient Solé et al. report for Saccharomyces cerevisiae is taken from a 2001 study [3] which in turn calculated the value based on high-throughput yeast two-hybrid data generated in 2000 [25]. In the intervening years the available protein interaction data has increased tremendously and has resulted in combined datasets with better coverage of the yeast interactome [30],[40]. It is known that an incomplete sample of a highly-clustered network produces a clustering coefficient lower than the actual network [48]. Therefore as the coverage of the sample increases, the clustering coefficient of the sample should increase as well, eventually reaching that of the actual network when the sample reaches total coverage. The observed clustering coefficients we report in Table 1 are significantly larger than 2.2×10^−2^, the observed clustering coefficient cited by Solé et al. So although the Solé et al. model produces clustering coefficients consistent with a 2000 dataset, it is low when compared to the more complete datasets available today.

A second discrepancy lies in the choice of random equivalent networks. Solé et al. note that their model produces a clustering coefficient roughly 10 times higher than random networks. The random networks they compare against are Erdős-Rényi random graphs which produce a Poisson degree distribution. This degree distribution is quite different than the power law degree distribution of protein interaction networks [49]. A more appropriate network comparison is against a network having an identical degree distribution, but with the edges randomized [50],[51]. Once equivalent random networks are employed, the reported 10-fold increase in clustering coefficient over random disappears. In fact, at *β* = 0.16 as published by Solé et al., the model produces clustering coefficients lower than equivalent random networks.

It is also useful to look beyond the topologies produced by theoretical models of homomeric duplication and neofunctionalization to the parameters of the models themselves. The Solé et al. model simulates neofunctionalization by forming de novo interactions between the newly-created duplicate and each of the other proteins in the network with probability *α*. If *α* is assigned a constant, gene duplicates will acquire an ever-increasing number of interacting partners as the network grows. For example, for *α* = 0.10, a duplicated gene in a 10-gene network will acquire one interacting partner on average. By the time the network grows to 100 genes, a gene duplicate will acquire 10 interacting partners on average. In order to maintain an average connectivity consistent with observed biological networks, *α* is adjusted downward as the network grows. Solé et al.'s duplication and diversification model calculates *α* as proportional to the inverse of the number of nodes currently in the network 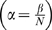 [7]. This parameterization is difficult to justify biologically. It requires a locally occurring phenomena to be cognizant of a global property of the system, in this case the total number of proteins.

By contrast, homomeric duplication models have no such restriction. The model introduced by Vázquez et al. [32] utilizes a simple constant for the probability of adding an interaction between the progenitor and progeny genes (i.e., the probability that a self-interacting protein was duplicated). In other words, gene duplicates are oblivious to the global state of the system.

Solé et al.'s neofunctionalization model and Vázquez et al.'s homomeric duplication model have also been compared in other venues. A study which used machine learning classification to compare seven network evolution models (including Vázquez et al. and Solé et al.) to the Drosophila melanogaster PIN found that the Vázquez et al. model produced networks closest to the Drosophila PIN [52]. Model validation of homomeric duplication was also performed by Ispolatov et al. [53] who found that the Vázquez et al. model generated clique distributions consistent with those observed in the Drosophila PIN.

The inability of models featuring neofunctionalization to produce a clustering coefficient greater than that of random equivalents, and the absence of a biologically rational method to produce de novo interactions during the evolution of the network argues against the prevalence of neofunctionalization. However, the neofunctionalization model need not be entirely abandoned. Though the neofunctionalization model has little evolutionary inferential efficacy, networks produced from the model have some topological value. The clustering coefficient is just one of several network measures used regularly in network analysis. Producing networks with characteristics consistent with observed PIN topologies is useful in biological network research, and models of both homomeric duplication and neofunctionalization continue to have utility in this regard [48],[54].

### Neofunctionalization Sensu Stricto

Although we have argued against the ubiquity of de novo interaction gain in protein interaction networks, this does not correspond to a denial of neofunctionalization. There are alternative evolutionary phenomena which may result in new functions, are relevant to protein interactions, and don't necessitate de novo interaction gain between extant proteins. New gene functions may arise through changes in interaction stochiometry or through the formation of new genes formed by exon shuffling, domain insertion, domain loss, domain shuffling, mobile elements, gene fusion, or gene fission [55],[56].

### Conclusion

Gene duplication is generally accepted as a key component of evolution, and protein interactions provide an attractive construct for studying the role of neofunctionalization, subfunctionalization, and homomeric duplication in evolution. Studies of protein interactions derived from empirical data and theoretical models of PIN evolution have regarded ubiquitous neofunctionalization as a requisite feature of post-duplication evolution. We have demonstrated assay limitations and the failure to recognize concurrent gene duplication and subfunctionalization underlie much of the literature which engender neofunctionalization as a prominent factor in protein interaction evolution. Furthermore, biologically implausible parameter requirements and distinctly non-biological clustering characteristics reduce the support theoretical models provide to a ubiquitous neofunctionalization argument.

It would be malapropos for us to assert that protein interaction evolution is absent of neofunctionalization. However, we believe de novo interaction gain is not as prevalent as previously thought. We have identified important factors which should be considered in any vetting of evolutionary interaction phenomenon before invoking neofunctionalization as a dominant mechanism.

## Methods

### Self Interactions in PDB

To get structural interactions, we first generated a non-redundant set of Saccharomyces cerevisiae proteins from the Protein Data Bank (PDB) [19]. The non-redundant set of protein complexes was identified in a manner similar to Levy et al. [57]. Specifically, for each structure in the PDB containing a yeast protein amino acid chain, create a simple undirected graph where each amino acid chain is an unlabeled node and interactions between different protein chains are edges. Group structures according to shared (isomorphic) graph topology. From these build subgroups according to shared sets of Pfam protein domains found in the complex. Further subdivide into subgroups containing the same set of proteins. One member from each of these subgroups is selected to be a non-redundant structure. The selected member is that with the X-ray crystallography structure having the greatest resolution.

We then cross-referenced this non-redundant structure set with interacting residue data gathered from version 21.0 of iPfam [20]. A protein was identified as self-interacting if there were two molecules (amino acid chains) of the protein within a complex that had interacting residues according to iPfam.

### Homomeric Interactions in the BRENDA Enzyme Database

Enzyme subunit composition was derived from the December, 2007 update of the BRENDA database [21]. BRENDA enzymes with subunit designations of homodimer, dimer, trimer, tetramer, hexamer, octamer, and nonamer were categorized as self-interacting. Monomers and heterodimers were categorized as non-self-interacting.

### Correcting for Subsequent Duplications

Gene dating (i.e., assigning genes to one of *T*_3_,*T*_2_,*T*_1_,*T*_0_ as shown in Figure 5) was derived from “orthogroup” gene trees from reference [28]. Gene duplications in the gene trees were associated with the phylogenetic nodes in which they occurred.

In Figure 6, black triangles are protein degrees after adjusting for more recent duplications. A black triangle aligned with *T*_3_ is the connectivity of a gene duplicate born in *T*_3_ after interactions with duplicates born during *T*_2_, *T*_1_, and *T*_0_ are removed. Black triangles in *T*_2_ have had interactions with duplicates born in *T*_1_ and *T*_0_ removed. Similarly, *T*_1_ black triangles have had interactions with *T*_0_ duplicates removed.

Duplications within each time period *T_i_* (*i* = 1,2,3), occurred sequentially over a period of evolutionary time and not concurrently. For a given duplication occuring in *T_i_*, on average one-half of the other duplications within *T_i_* occurred subsequent to the given duplication. Therefore, in addition to removing interactions in subsequent time periods as specified above, duplications occurring in the same time period multiplied by 0.5 are also removed.

Singleton genes, that is genes not associated with any duplication event, are considered to have birthdays preceding *T*_3_ in Figure 5. Singletons interacting with plotted proteins are included in the degree tally, but are not themselves plotted because, by definition, they did not arise during *T*_3_,*T*_2_, or *T*_1_.

Each duplication has a progenitor, the ancestral gene, and a progeny, the gene born of the duplication. An issue to be addressed is which gene is the progenitor and which is the progeny. In some cases this is unambiguous. For example, an orthogroup may have three paralogous members: *P_A_*, *P_B_*, and *P_C_*. A common ancestor would have a single gene: *P_ABC_*. During evolution a duplication event would produce an extant progeny gene (*P_A_*) and an ancestral progenitor gene (*P_BC_*). However, the vast majority of orthogroups contain only two genes. In these cases the duplication event produces two extant genes, making the assignment of progenitor and progeny ambiguous.

To address this ambiguity, extant genes pairs produced from duplication events were randomly assigned “progenitor” and “progeny” labels. This random assignment was repeated 100 times and the protein connectivity of the 100 progeny assignments both before and after accounting for subsequent duplications was averaged and plotted as shown in Figure 6.

Duplicate pairs in which both members had degree zero were omitted from the analysis.

### Duplicates Enriched in Paralogs

All duplication events resulting in two extant genes were paired and dated as described above. For each paralogous protein pair born in *T*_3_, the non-redundant set of their neighbors was identified. Paralogous pairs born in *T*_2_,*T*_1_, and *T*_0_ were counted as neighbors of the *T*_3_ pair if both paralogs of the younger pair were part of the non-redundant set. Paralogous pairs born in *T*_3_ were counted at half for the reasons specified above. The equivalent process was used to identify paralogous neighbors of pairs born in *T*_2_ and *T*_1_. The P-value represents the number of times a random network with identical topology is at least as enriched in paralogs. To compute the P-value, the gene lables on the network were randomized 10^6^ and the same computation done. As the P-value indicates, none of the randomized networks were as enriched as the empirical networks.

### Equivalent Random Networks

Equivalent random networks were generated in order to derive clustering coefficients. Because self-interactions are not included in calculating the clustering coefficient, they were ignored for purposes of creating the random networks. The equivalent random networks used in Table 1 and Figure 9 were generated by rewiring links while preserving the degree distribution [51]. At each iteration a pair of edges were selected at random and one end from each edge was swapped. If the swap created a duplicate edge or a self-interaction the swap was aborted and the next iteration begun. The number of iterations performed was 100*E* where *E* is the number of edges in the network.

### Network Enumeration

First note that any connected network with *N* nodes must have a minimum of *N*−1 edges (i.e., a tree). All non-isomorphic connected networks with *N* nodes were determined in two stages. In stage one, a set of *N*-node trees was built from *N*−1-node trees established in the previous iteration by adding a node and testing for isomorphism each network generated by adding an edge between the new node and each existing node.

Stage 2 follows similarly by iteratively testing networks for isomorphism by adding a single edge to existing *N*−*node* networks until *N*(*N*−1)/2 edges is reached (i.e., the number of edges in a completely connected *N*-node network).

The algorithm begins with the two possible 3-node networks, *C*_3_ and *P*_3_. Isomorphism is a computationally expensive process. Therefore, isomorphism comparisons were first pre-screened by only evaluating networks with an identical number of edges, nodes, degree distribution, and 2-hop distribution. The algorithm as described in reference [58] was used to determine network isomorphism. Table 2 shows cumulative Δ*C* of simple duplication and homomeric duplication of the enumerated networks as the number of nodes increases.

### Neofunctionalization and Homomeric Duplication Networks

For the plots in Figure 9, each network began with a 100-node Erdős-Renyí seed graph. The seed graph was generated by randomly adding edges between the *N*(*N*−1)/2, *N* = 100 node pair combinations with a probability *p* = 0.04. We ensured homogeneity by using the same seed graph for each network. Each simulation included simple duplication and subfunctionalization. Figure 9A added neofunctionalization, while Figure 9B added homomeric duplication to simple duplication and subfunctionalization.

Simple duplication is defined as randomly selecting an existing node in the network, identifying the set of neighbors the selected node interacts with, and adding a new node to the network which interacts with an identical set of neighbors. Subfunctionalization is defined as removing each interaction from the newly-added node with a given probability. Neofunctionalization is defined as adding an interaction from the newly-added node to each existing node in the network with a given probability *β*. Homomeric duplication is defined as adding an interaction between the randomly-selected node (i.e., the progenitor) and the newly-added node (i.e., the progeny) with a given probability. Newly-added nodes having no interacting partners after going through the relevant evolutionary processes were discarded.

Simulated networks were evolved until they reached 5794 nodes, the putative number of yeast genes. Each line plotted in the figure was based on the mean clustering coefficient of 100 networks for each of 80 loss probabilities: [0.20,0.21,…,0.99]. That is, each line is the result of 80×100 = 8000 generated networks. In the neofunctionalization plot probabilities 0.20 thru 0.39 were not calculated for *β* = 50 nor were probabilities 0.20 thru 0.22 for *beta* = 16 due to prohibitive runtime and/or overflow errors in the 32-bit numbers used to store the number of triangles and triples in the growing networks.

## Supporting Information

Text S1 A proof that the change in clustering coefficient is always greater for a homomeric duplication than for an equivalent simple (non-homomeric) duplication.
(0.07 MB PDF)
