# Supplementary material for: Questioning the Ubiquity of Neofunctionalization
Source: PLoS Comput Biol. 2009 Jan 2;5(1):e1000252. doi: 10.1371/journal.pcbi.1000252 (PMC2597716; doi:10.1371/journal.pcbi.1000252)
Supplement: Text S1 — A proof that the change in clustering coefficient is always greater for a homomeric duplication than for an equivalent simple (non-homomeric) duplication. (0.07 MB PDF) [file pcbi.1000252.s001.pdf]

Text S1: Supporting Information for Questioning the ubiquity of neofunctionalization

November 11, 2008

Proof: $\Delta C_{\mathbf{homomer}} > \Delta C_{\mathbf{simple}}$ for networks with at least one triangle

The clustering coefficient is defined as:

$$C = \frac{3T}{\Gamma}$$

where T is the number of triangles and Γ is the number of connected triples. A triangle is three fully-connected nodes and a connected triple is a node connected to an unordered pair of other nodes (i.e., a path of length 2) [1]. For simplicity, we associate a connected triple with its middle node. The number of connected triples is calculated for a network with N proteins by:

$$\Gamma = \sum_{i=1}^{N} \frac{k_i(k_i-1)}{2}$$

where k_i is the number of neighbors, or degree, of protein i.

For a single, simple duplication, the new clustering coefficient is calculated as:

$$\frac{3(T+t_p)}{\Gamma + \gamma_p + \sum k_g}$$

The progeny protein acquires the same number of triangles and connected triples as the progenitor (t_p and $\gamma_p = \frac{k_p(k_p-1)}{2}$ respectively). Additionally, each of the progenitor's neighbors $g = \{1..k_p\}$ gain k_g connected triples due to the additional edge the progeny contributes to each neighbor (Figure 7): That is, the connected triples gained by all neighbors due to simple duplication equal $\sum_{g=1}^{k_p} k_g$.

If the duplicated protein is self-interacting, it acquires all of the triangles and connected triples of its non-self-interacting counterpart, plus k_p additional triangles and $2k_p$ additional connected triples (Figure 7):

$$\frac{3(T+t_p+k_p)}{\Gamma + \gamma_p + \sum k_g + 2k_p}$$

To prove: the post-duplication clustering coefficient is greater if the duplicated protein is homomeric.

$$\frac{3(T + t_p + k_p)}{\Gamma + \gamma_p + \sum k_g + 2k_p} \overset{?}{>} \frac{3(T + t_p)}{\Gamma + \gamma_p + \sum k_g}$$

establish a common denominator:

$$(T + t_p + k_p)(\Gamma + \gamma_p + \sum k_g) \overset{?}{>} (T + t_p)(\Gamma + \gamma_p + \sum k_g + 2k_p)$$

multiply out and remove like terms T and t_p:

$$k_p\Gamma + k_p\gamma_p + k_p \sum k_g \overset{?}{>} 2Tk_p + 2t_pk_p$$

finally, divide out k_p:

$$\Gamma + \gamma_p + \sum_{g=1}^{k_p} k_g \overset{?}{>} 2T + 2t_p$$

By the definition of the clustering coefficient, $\Gamma \geq 3T$. In the non-trivial case of networks with at least one triangle, $\Gamma > 2T$. For the remaining terms note that $\sum k_g \geq k_p$ since each neighbor of p has degree at least one (p is a neighbor). Additionally, each progenitor triangle, t_p, increases the degree of two neighbors by 1 since an edge must connect two neighbors to form the triangle. Therefore, $\sum k_g \geq 2t_p + k_p$. The inequality holds:

$$\Delta C_{\text{homomer}} > \Delta C_{\text{simple}}$$

References

[1] Newman ME (2001) The structure of scientific collaboration networks. Proc Natl Acad Sci U S A 98: 404–409.
